# Role of inflammation in previously untreated macular edema with branch retinal vein occlusion

**DOI:** 10.1186/1471-2415-14-67

**Published:** 2014-05-18

**Authors:** Hidetaka Noma, Tatsuya Mimura, Katsunori Shimada

**Affiliations:** 1 Department of Ophthalmology, Yachiyo Medical Center, Tokyo Women’s Medical University, 477-96, Owada-shinden, Yachiyo, Chiba, Japan; 2 Department of Ophthalmology, Medical Center East, Tokyo Women’s Medical University, Tokyo, Japan; 3 Department of Biostatistics, STATZ Institute Inc., Tokyo, Japan

## Abstract

**Background:**

The association of inflammatory factors and the aqueous flare value with macular edema in branch retinal vein occlusion (BRVO) patients remains unclear. The relationship between the aqueous flare value and the vitreous fluid levels of vascular endothelial growth factor (VEGF), interleukin (IL)-6, monocyte chemotactic protein (MCP)-1, soluble intercellular adhesion molecule 1 (sICAM-1), and soluble VEGF receptor-2 (sVEGFR-2) was evaluated to investigate the role of inflammation in BRVO associated with macular edema. Aqueous flare values and the vitreous levels of VEGF, IL-6, MCP-1, sICAM-1, and sVEGFR-2 were compared between previously untreated patients with BRVO and patients with macular hole (MH).

**Methods:**

Vitreous samples were obtained from 45 patients during vitreoretinal surgery (28 patients with BRVO and 17 with MH), and the levels of VEGF, IL-6, MCP-1, sICAM-1, and sVEGFR-2 were measured by enzyme-linked immunosorbent assay. Retinal ischemia was evaluated by measuring the area of capillary non-perfusion using fluorescein angiography and the Scion Image program. Aqueous flare values were measured with a laser flare meter and macular edema was examined by optical coherence tomography.

**Results:**

The median aqueous flare value was significantly higher in the BRVO group (12.1 photon counts/ms) than in the MH group (4.5 photon counts/ms, *P* < 0.001). There were significant correlations between the aqueous flare value and the vitreous levels of VEGF, IL-6, MCP-1, and sICAM-1 in the BRVO group (ρ = 0.54, *P* = 0.005; ρ = 0.56, *P* = 0.004; ρ = 0.52, *P* = 0.006; and ρ = 0.47, *P* = 0.015, respectively). The aqueous flare value was also significantly correlated with the foveal thickness in the BRVO group (ρ = 0.40, *P* = 0.037).

**Conclusions:**

Inflammation may induce an increase of vascular permeability and disrupt the blood-aqueous barrier via release of inflammatory factors (VEGF, IL-6, MCP-1, and sICAM-1) in BRVO patients with macular edema.

## Background

Branch retinal vein occlusion (BRVO) is a common retinal vascular disease that is frequently associated with macular edema, which is the chief reason for visual impairment in BRVO patients. Occlusion of blood vessels causes bleeding and accumulation of serous fluid, which leading to edema. It is considered that inflammation develops due to damage arising from this process. Intravitreal injection of bevacizumab (a monoclonal antibody targeting vascular endothelial growth factor (VEGF)) or ranibizumab (an Fab fragment that binds and neutralizes all isoforms of VEGF-A) has been reported to improve macular edema in patients with BRVO [1,2]. However, some patients have persistent macular edema after intravitreal treatment with bevacizumab or ranibizumab [3,4], suggesting that other vasoactive factors may contribute to macular edema in addition to VEGF. We recently reported that intraocular levels of inflammatory factors, which are expressed by migrating leukocytes, glia cells, and vascular endothelial cells, were significantly correlated with the severity of macular edema in BRVO patients [5,6], suggesting an important role for inflammation in the occurrence of macular edema. This hypothesis is supported by the results of the Standard Care vs Corticosteroid for Retinal Vein Occlusion (SCORE) study, which showed that intravitreal injection of triamcinolone acetonide improved visual acuity and macular edema after 12 months in patients with BRVO [7]. Moreover, a larger non-perfused area is associated with higher intraocular levels of these inflammatory factors [5,6]. However, even some patients without any nonperfused retinal areas (nonischemic BRVO) have increased levels of inflammatory factors, suggesting that inflammation may promote macular edema in patients with nonischemic BRVO.

Laser flare photometry is an objective method for evaluating the aqueous flare and cells, and it may allow quantitative assessment of anterior chamber inflammation and breakdown of the blood–aqueous barrier [8]. It has also been reported that the aqueous flare (photon counts/ms) is correlated with the aqueous humor protein content (mg/ml), including total protein, albumin, and immunoglobulin G [9,10]. Furthermore, it was reported that the aqueous flare value is significantly higher in patients with retinal vein occlusion (RVO) than in normal controls [11,12], suggesting that an increased flare value reflects disruption of the blood-ocular barrier (both the blood-retinal barrier and the blood-aqueous barrier) and inflammation in eyes with RVO. In fact, the ratio of aqueous cells to the aqueous flare value is approximately 1:3 in BRVO patients [12]. However, the association of inflammatory factors and the flare value with macular edema in BRVO patients remains unclear. We previously investigated the correlation of inflammatory factors with retinal changes [13], while the correlation with the aqueous flare was investigated in this study. We also recently reported on the correlation of inflammatory factors with the aqueous flare in patients with CRVO [14], which is a pathologically different condition from BRVO. Therefore, we evaluated the relations between macular edema and indicators of inflammation (aqueous flare value and vitreous fluid levels of interleukin (IL)-6, monocyte chemotactic protein (MCP)-1, VEGF, intercellular adhesion molecule (ICAM)-1, and VEGF receptor (VEGFR)-2) in patients with BRVO and macular edema.

## Methods

### Subjects

Undiluted vitreous fluid samples were harvested at the start of pars plana vitrectomy (PPV) after written informed consent was obtained from each subject following an explanation of the purpose and potential adverse effects of the procedure. This study was performed in accordance with the Helsinki Declaration of 1975 (1983 revision). The institutional review boards of Tokyo Women’s Medical University approved the protocol for collection and testing of vitreous fluid samples. Consecutive previously untreated patients with BRVO who presented between June 2008 and September 2011 were screened using the criteria listed below and vitreous fluid samples were obtained from the 28 patients who were enrolled. The indications for PPV were: (1) clinically detectable macular edema or cystoid macular edema persisting for more than 3 months, (2) best-corrected visual acuity worse than 20/40 using the Snellen visual acuity chart, (3) onset of symptoms within 1 month before presentation, and (4) foveal thickness >250 μm by OCT. In addition, the subjects were limited to patients who could fix on the central landmark during optical coherence tomography. Exclusion criteria were (1) previous ocular surgery or intravitreous injection of anti-VEGF agents or triamcinolone acetonide, (2) diabetes mellitus with diabetic retinopathy, (3) previous retinal photocoagulation, (4) iris rubeosis, and (5) a history of ocular inflammation or vitreoretinal disease. Best-corrected visual acuity was converted to the logarithm of the minimum angle of resolution (LogMAR).

Twenty-eight BRVO patients and 17 control patients with nonischemic ocular disease were enrolled (Table 1). Vitreous fluid samples were also obtained from the 17 controls, who all had idiopathic macular hole (MH group). None of the patients in the MH group had proliferative vitreoretinopathy. PPV was performed at Tokyo Women’s Medical University.

**Table 1:** Clinical and laboratory characteristics of the BRVO group and the macular hole group

	**BRVO group**	**Macular hole group**	***P *** **value**
Number^†^	28	17	
Gender (Male/Female)	17/11	8/9	0.371
Age (yr)	71.2 ± 7.8^‡^	68.9 ± 6.0^‡^	0.304
Blood pressure (mmHg)			
Systolic	135 ± 15^‡^	115 ± 14^‡^	<0.001
Diastolic	80 ± 10^‡^	71 ± 9^‡^	0.004
Hypertension	17	2	0.001
Hyperlipidemia	7	4	0.911
Duration of BRVO (months)	4.7 ± 2.2^‡^	-	-
Aqueous flare (photon counts/ms)	12.1 [8.2–14.8]^‡^	4.5 [3.9–6.1] ^‡^	<0.001
Nonperfused area (disc area)	44.7 ± 32.5^‡^	-	-
Foveal thickness (μm)	557 ± 135^‡^	-	-

^†^Number of patients with data. ^‡^Mean ± standard deviation (SD).

BRVO = branch retinal vein occlusion.

### Fundus findings

The fundus was examined preoperatively by biomicroscopy with a fundus contact lens and by standard fundus color photography. In addition, fluorescein angiography was performed with a Topcon TRC-50EX fundus camera, an image-net system (Tokyo Optical Co. Ltd., Japan), and a preset lens with a slit-lamp.

Preoperative fundus findings were recorded for each subject. A masked grader independently assessed retinal perfusion status or ischemic retinal vascular occlusion by examination of fluorescein angiograms. The ischemic region of the retina was measured using the public domain Scion Image program, as reported previously [5,6].

Optical coherence tomography (OCT) was performed in each subject within 1 week before vitrectomy, employing an instrument from Zeiss-Humphrey Ophthalmic Systems (Zeiss Stratus OCT3, Carl Zeiss Meditec, Dublin, CA, USA). The fundus was scanned with the measuring beam focused on horizontal and vertical planes crossing the center of the fovea, which was located by examination of the fundus photograph and by each patient’s fixation. Cross-sectional images were collected by a single experienced examiner, who continued each examination until highly reproducible scans were obtained. The thickness of the central fovea was defined as the distance between the inner limiting membrane and the retinal pigment epithelium (including any serous retinal detachment), and was automatically measured by computer software. The thickness of the neurosensory retina was defined as the distance between the inner and outer neurosensory retinal surfaces. The average preoperative foveal thickness was 557 ± 135 μm, with a range of 292 to 834 μm.

### Aqueous flare measurement

The aqueous flare was measured with a laser flare meter (FC-600, Kowa Co. Ltd., Tokyo, Japan), as described previously [8]. The sensitivity and reproducibility of this method have been confirmed by a number of studies [8,11,12]. Measurements were performed within 1 week before treatment. Flare values were measured at 30 minutes after dilation of the pupil with 0.5% tropicamide and 5% phenylephrine hydrochloride. Two different examiners obtained five measurements from each eye and the results were averaged after excluding all measurements with artefacts. The laser flare meter that was employed could measure values up to 500 photon counts/ms in patients without hypopium or hyphema. We performed standardization before each test. If deviation was found, we adjusted the device by setting the zero value with a built-in calibration function.

### Sample collection

Samples of undiluted vitreous fluid (0.3 – 0.7 ml) were collected at the start of vitrectomy by aspiration into a 1 ml syringe attached to the vitreous cutter before commencing the intravitreal infusion of balanced salt solution. After collection, the vitreous samples were immediately transferred into sterile tubes and were stored frozen at -80°C within 5 minutes.

### Measurement of VEGF, IL-6, MCP-1, sICAM-1, and sVEGFR-2

The levels of VEGF, IL-6, MCP-1, soluble ICAM-1 (sICAM-1), and soluble VEGFR-2 (sVEGFR-2), were measured in vitreous fluid samples from the same eye by enzyme-linked immunosorbent assay using kits for human VEGF, IL-6, MCP-1, sICAM-1, and sVEGFR-2 (VEGF, IL-6, MCP-1, and sVEGFR-2: R&D Systems, Minneapolis, MN, USA; sICAM-1: Bender Med Systems, Burlingame, CA, USA) [5,6,13]. The VEGF kit was able to detect 2 of the 4 VEGF isoforms (VEGF_121_ and VEGF_165_), probably because these 2 shorter isoforms are secreted and the 2 longer isoforms are cell-associated. Each assay was performed according to the manufacturer’s instructions. The standard solution (100 μl for VEGF or sICAM-1, 150 μl for IL-6, 200 μl for MCP-1, and 100 μl for sVEGFR-2) and the sample (100 μl for VEGF, 150 μl for IL-6, 40 μl for MCP-1, 10 μl for sICAM-1, and 10 μl for sVEGFR-2) were added to the wells of a 96-well plate coated with the relevant monoclonal antibody. After incubation, the plate was washed and an enzyme-labeled antibody was added. After further incubation, the plate was washed again and the substrate was added. The reaction was stopped by adding the stop solution after color had been developed, and the optical density was determined at 450 and 620 nm using an absorption spectrophotometer (Titertek Multiscan MCC/340; ICN, Tokyo, Japan). The levels of these factors in the vitreous fluid samples and plasma were within the detection ranges of the assays, with the minimum detectable concentration was 15.6 pg/ml for VEGF (intra-assay coefficient of variation (CV), 5.2%; inter-assay CV, 6.6%), 0.156 pg/ml for IL-6 (intra-assay CV, 5.3%; inter-assay CV, 6.5%), 62.5 pg/ml for MCP-1 (intra-assay CV, 4.6%; inter-assay CV, 5.8%), 3.3 ng/ml for sICAM-1 (intra-assay CV, 5.5%; inter-assay CV, 7.6%), and 78.1 pg/ml for sVEGFR-2 (intra-assay CV, 5.4%; inter-assay CV, 6.8%).

### Statistical analysis

All analyses were performed with SAS System 9.1 software (SAS Institute Inc., Cary, North Carolina, USA). Data are presented as the mean ± SD or as the median with the interquartile range or frequency. Student’s *t*-test was employed to compare normally distributed unpaired continuous variables between the two groups and the Mann–Whitney U test was used for variables with a skewed distribution. To examine the relations among vitreous levels of VEGF, IL-6, MCP-1, sICAM-1, or sVEGFR-2, the aqueous flare value, the nonperfused area, and the foveal thickness, Spearman’s rank-order correlation coefficients and a multiple linear regression model were used. Two-tailed p values of less than 0.05 were considered to indicate a statistically significant difference.

## Results

The BRVO group (17 men and 11 women) was aged 71.2 ± 7.8 years (mean ± SD), while the MH group (8 men and 9 women) was aged 68.9 ± 6.0 years (Table 1). The mean duration of BRVO was 4.7 ± 2.2 months (range: 3 – 10 months) (Table 1). Seventeen of the 28 BRVO patients (61%) had hypertension and seven patients (25%) had hyperlipidemia (Table 1). Mean BCVA was logMAR 0.77 ± 0.40. There was no significant correlation between BCVA and the aqueous flare value (ρ = 0.19, *P* = 0.320). There were also no significant correlations between BCVA and the vitreous levels of VEGF, IL-6, MCP-1, sICAM-1, and sVEGFR-2 (ρ = 0.33, *P* = 0.080; ρ = 0.18, *P* = 0.353; ρ = 0.17, *P* = 0.375; ρ = -0.16, *P* = 0.409; and ρ = -0.06, *P* = 0.747, respectively).

**Figure 1:**
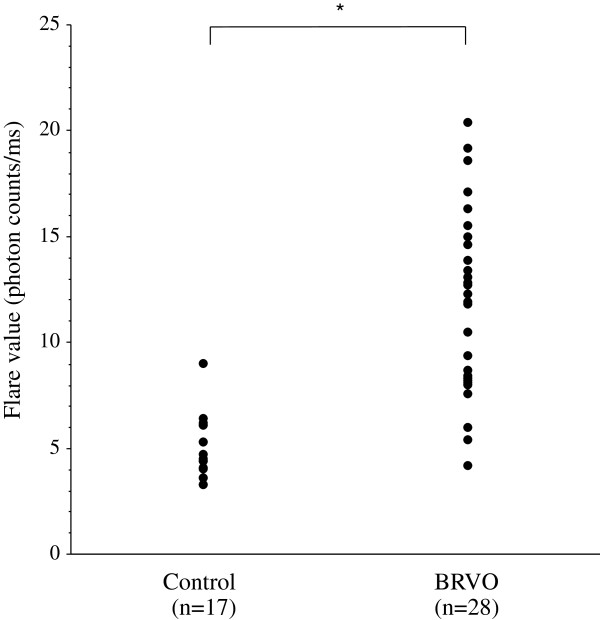
**Aqueous flare value in the control group (macular hole) and in the patients with branch retinal vein occlusion (BRVO) and macurla edema.** The flare value was significantly higher in the BRVO group than in the MH group (*P < 0.001).

**Figure 2:**
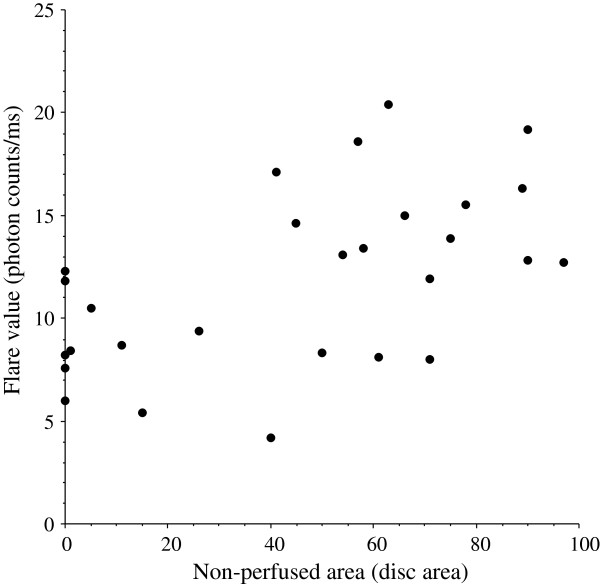
**Correlation between the aqueous flare value and the nonperfused area of the retina.** The aqueous flare value was significantly correlated with the nonperfused area of the retina in BRVO group (ρ = 0.55, *P* = 0.002).

The aqueous flare value (median [interquartile range]) was significantly higher in the BRVO group (median: 12.1 photon counts/ms [interquartile range: 8.2 – 14.8]) than in the MH group (4.5 photon counts/ms [3.9 – 6.1], *P* < 0.001) (Figure 1). The aqueous flare value was significantly correlated with the nonperfused area of the retina in the BRVO group (ρ = 0.55, *P* = 0.002) (Figure 2).

The vitreous fluid concentration of VEGF was significantly higher in the BRVO group (754 pg/ml [59.5-1760]) compared with the MH group (15.6 pg/ml [15.6-23.1]) (*P* < 0.001) (Table 2). Likewise, the vitreous level of IL-6 was significantly higher in the BRVO group (10.0 pg/ml [5.43-22.5]) than in the MH group (1.04 pg/ml [0.60-1.17], *P* < 0.001), as was the vitreous level of MCP-1 (BRVO group: 1220 pg/ml [921–1925]; MH group: 411 pg/ml [384–582], *P* < 0.001) (Table 2). Furthermore, the vitreous level of sICAM-1 was significantly higher in the BRVO group (9.40 ng/ml [6.45-14.3]) than in the MH group (4.00 ng/ml [3.37-4.92], *P* < 0.001), as was the vitreous level of sVEGFR-2 (BRVO group: 1600 pg/ml [1007–1990]; MH group: 840 pg/ml [617–1067], *P* < 0.001) (Table 2). There were significant correlations between the nonperfused area of the retina and the vitreous fluid levels of VEGF, IL-6, MCP-1, and sICAM-1 in the BRVO group (ρ = 0.72, *P* < 0.001; ρ = 0.59, *P* = 0.002; ρ = 0.41, *P* = 0.034; and ρ = 0.38, *P* = 0.043, respectively) (Table 3). In order to clarify which factor (VEGF, IL-6, MCP-1, sICAM-1, or sVEGFR-2) was most closely correlated with the nonperfused area of the retina, multiple linear regression analysis with stepwise selection of variables was performed. This analysis showed that the vitreous level of VEGF was most strongly correlated with the nonperfused area of the retina. In the BRVO group, vitreous fluid levels of VEGF, IL-6, MCP-1, sICAM-1, and sVEGFR-2 were significantly correlated with the foveal thickness (ρ = 0.50, *P* = 0.009; ρ = 0.54, *P* = 0.003; ρ = 0.43, *P* = 0.025; ρ = 0.49, *P* = 0.008; and ρ = 0.38, *P* = 0.044, respectively) (Table 3).

**Table 2:** Vitreous fluid levels of factors in the two groups

	**BRVO group**	**Macular hole group**	***P *** **value**
VEGF (pg/ml)	754 [59.5–1760]	15.6 [15.6–23.1]	<0.001
IL-6 (pg/ml)	10.0 [5.43–22.5]	1.04 [0.60–1.17]	<0.001
MCP-1 (pg/ml)	1220 [921–1925]	411 [384–582]	<0.001
sICAM-1 (ng/ml)	9.40 [6.45–14.3]	4.00 [3.37–4.92]	<0.001
sVEGFR-2 (pg/ml)	1600 [1007–1990]	840 [617–1067]	<0.001

Values are the median [interquartile range]. BRVO = branch retinal vein occlusion; VEGF = vascular endothelial growth factor; IL-6 = interleukin-6; MCP-1 = monocyte chemotactic protein-1; soluble intercellular adhesion molecule 1 = sICAM-1; soluble vascular endothelial growth factor receptor-2 = sVEGFR-2.

**Table 3:** Correlations among the variables

		**VEGF**	**IL-6**	**MCP-1**	**sICAM-1**	**sVEGFR-2**
Aqueous flare	γ=ρ value	0.54	0.56	0.52	0.47	0.20
	*P* value	0.005	0.004	0.006	0.015	0.295
Nonperfused area	γ=ρ value	0.72	0.59	0.41	0.38	0.18
	*P* value	<0.001	0.002	0.034	0.043	0.343
Foveal thickness	γ=ρ value	0.50	0.54	0.43	0.49	0.38
	*P* value	0.009	0.003	0.025	0.008	0.044

VEGF = vascular endothelial growth factor; IL-6 = interleukin-6; MCP-1 = monocyte chemotactic protein-1; soluble intercellular adhesion molecule 1 = sICAM-1; soluble vascular endothelial growth factor receptor-2 = sVEGFR-2; γ=ρ = correlation coefficient.

**Figure 3:**
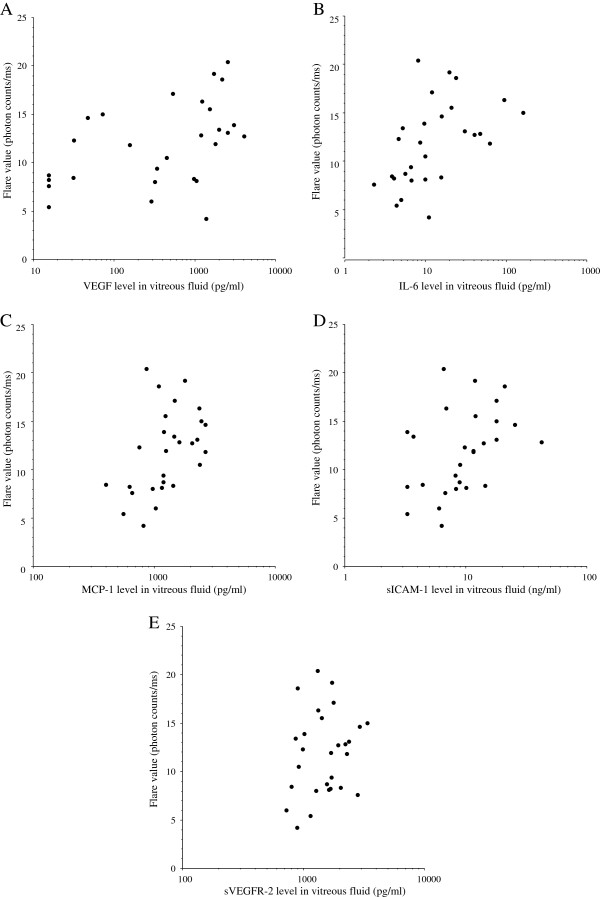
**Correlations between the aqueous flare value and the vitreous fluid levels of vascular endothelial growth factor (VEGF), interleukin (IL)-6, monocyte chemotactic protein (MCP)-1, soluble intercellular adhesion molecule 1 (sICAM-1), and soluble VEGF receptor-2 (sVEGFR-2).** There were significant correlations between the flare value and vitreous levels of VEGF **(A)**, IL-6 **(B)**, MCP-1 **(C)**, sICAM-1 **(D)**, and sVEGFR-2 **(E)** in the BRVO group (ρ = 0.54, *P* = 0.005; ρ = 0.56, *P* = 0.004; ρ = 0.52, *P* = 0.006; ρ = 0.47, *P* = 0.015; and ρ = 0.20, *P* = 0.295, respectively).

**Figure 4:**
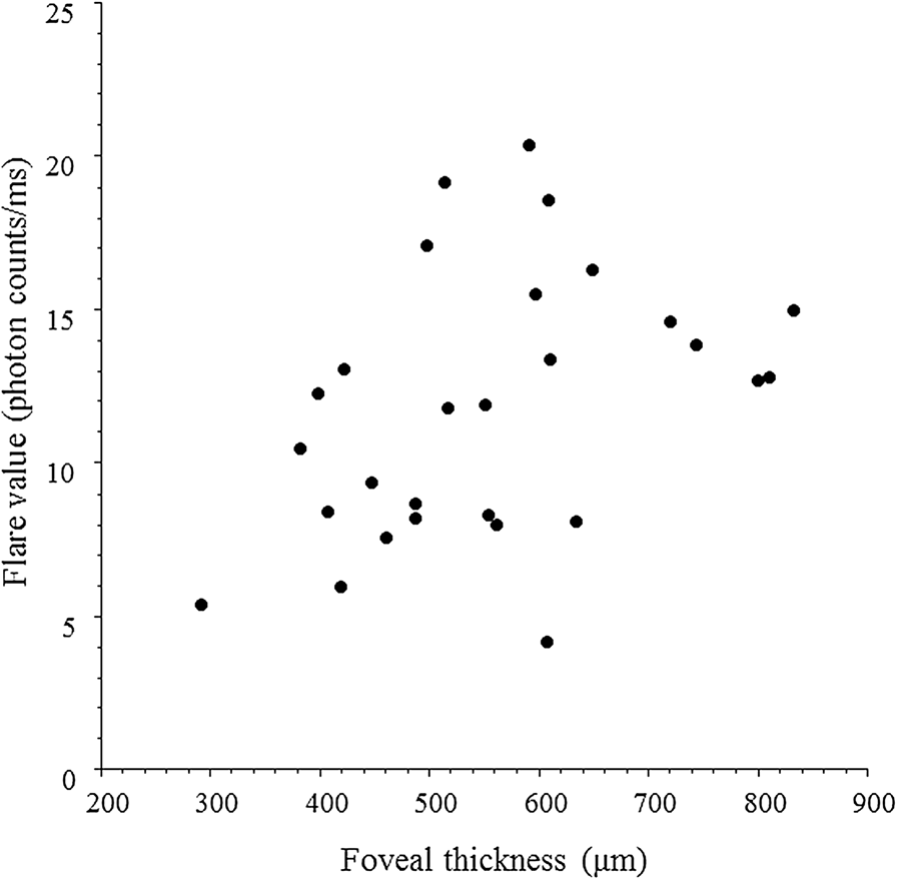
**Correlation between the aqueous flare value and the foveal thickness.** The aqueous flare value was significantly correlated with the foveal thickness in the BRVO group (ρ = 0.40, *P* = 0.037).

There were significant correlations between the aqueous flare value and the vitreous fluid levels of VEGF, IL-6, MCP-1, and sICAM-1 in the BRVO group (ρ = 0.54, *P* = 0.005; ρ = 0.56, *P* = 0.004; ρ = 0.52, *P* = 0.006; and ρ = 0.47, *P* = 0.015, respectively) (Figure 3A-D) (Table 3). However, there was no significant correlation between the aqueous flare value and the vitreous fluid level of sVEGFR-2 (ρ = 0.20, *P* = 0.295) (Figure 3E) (Table 3). To clarify which factor (VEGF, IL-6, MCP-1, sICAM-1, or sVEGFR-2) was most closely correlated with the aqueous flare value, multiple linear regression analysis with stepwise selection of variables was performed. This analysis revealed that VEGF was most strongly correlated with the aqueous flare value. In addition, the aqueous flare value was significantly correlated with the foveal thickness in the BRVO group (ρ = 0.40, *P* = 0.037) (Figure 4).

The BRVO patients were divided into fresh BRVO (onset < 7 months before the study) and older BRVO subgroups for further analysis [15]. There was no significant difference of the aqueous flare value between the fresh BRVO subgroup (n = 21) and the older BRVO subgroup (n = 7) (*P* = 0.441). There were also no significant differences with regard to the vitreous levels of the five factors (VEGF, IL-6, MCP-1, sICAM-1, and sVEGFR-2) between the fresh and older subgroups (*P* = 0.915; *P* = 0.577; *P* = 0.524; *P* = 0.490; and *P* = 0.691, respectively).

## Discussion

The Branch Vein Occlusion Study demonstrated the effectiveness of argon laser photocoagulation for BRVO, but it was recommended that this should not be performed within 3 months after the onset since spontaneous improvement can occur during this period [16]. It has also been reported that the absence of posterior vitreous detachment can contribute to persistent macular edema in patients with retinal vascular occlusion [17], while Saika et al. reported the effectiveness of vitrectomy combined with surgical posterior vitreous detachment for macular edema in BRVO patients [18]. Moreover, it has been reported that PPV contributes to an increase of oxygen tension in the inner retina [19,20]. If the retinal oxygen tension is increased by PPV, macular edema would improve because the increase of oxygen tension reduces VEGF production and thus decreases vascular permeability. An increase of oxygen tension would also alleviate autoregulatory arteriolar vasoconstriction and reduce the hydrostatic pressure in the retinal capillaries and venules, which would decrease water flux from the vascular compartment to the tissue compartment and lessen edema according to Starling’s law. Consistent with this hypothesis, it has been reported that vitrectomy improves both functional and tomographic outcomes in BRVO patients with macular edema [21,22]. Accordingly, we performed vitrectomy in patients who had clinically detectable diffuse macular edema or cystoid macular edema at more than 3 months after the occurrence of BRVO.

The present study demonstrated that the aqueous flare value (an index of inflammation) was significantly higher in the BRVO group than in the MH group, and the flare value was also significantly correlated with the nonperfused area of the retina in the BRVO group. In addition, the vitreous fluid levels of VEGF, IL-6, MCP-1, and sICAM-1 were significantly correlated with the nonperfused area of the retina and with the foveal thickness in the BRVO group. We previously reported that there were no correlations between the intraocular and plasma levels of various cytokines, which suggested that the intraocular elevation of cytokines was not related to breakdown of the blood-retinal barrier and/or intraocular bleeding [5,6,13]. These findings suggest that both ischemia and inflammation may have an important role in breakdown of the blood-retinal barrier and development of macular edema.

Moreover, we found a significant correlation between the aqueous flare value and the vitreous fluid levels of VEGF, IL-6, MCP-1, and sICAM-1 in the BRVO group. Miyake et al. [11] performed anterior chamber and vitreous fluorophotometry in patients with RVO, and observed that the fluorescein concentration was only increased in the anterior chamber and the posterior vitreous, whereas its level in the middle vitreous was low or normal. Accordingly, they concluded that the increase of the aqueous flare in RVO patients mainly reflects disruption of the blood-aqueous barrier. Thus, the blood-aqueous barrier may be abnormal in the anterior segment of eyes with RVO. Furthermore, Virdi et al. [23] found an increase of fluorescein in the aqueous humor and vascular leakage from the iris on fluorescein angiography in patients with major branch RVO or central RVO who had no evident iridal abnormalities or rubeosis. Fluorescein leakage from the iridal vessels was also found in monkeys with experimental RVO before the occurrence of neovascularisation of the iris [23]. These findings and our results suggest that the aqueous flare value may be increased by leakage of protein from iridal vessels after disruption of the blood-aqueous barrier due to the effects of inflammatory factors such as VEGF, IL-6, MCP-1, and sICAM-1.

Interestingly, we found a significant correlation between the aqueous flare value (an index of inflammation) and the foveal thickness in the BRVO group (Figure 4). This finding is supported by a report that the incidence of cystoid macular edema is associated with the severity of blood-aqueous barrier disruption [24], and also suggests that inflammation may promote macular edema in BRVO patients. As mentioned above, we found a significant correlation between the foveal thickness and the vitreous fluid levels of VEGF, IL-6, MCP-1, and sICAM-1 in the BRVO group (Table 2). Therefore, the correlation between aqueous flare and foveal thickness in this group might suggest that these inflammatory factors (VEGF, IL-6, MCP-1, and sICAM-1) are involved in the development of macular edema by increasing vascular permeability and/or promoting diapedesis of leukocytes. Taken together, these findings suggest that, in addition to ischemia, inflammation is involved in the increase of vascular permeability and disruption of the blood-aqueous barrier via release of inflammatory factors (VEGF, IL-6, MCP-1, and sICAM-1) in BRVO patients with macular edema. However, the present study was small, so a larger prospective investigation will be required to confirm the influence of inflammation on macular edema associated with BRVO.

In the present study, multiple linear regression analysis showed that VEGF was most strongly correlated with the aqueous flare value among the five vitreous factors (VEGF, IL-6, MCP-1, sICAM-1, and sVEGFR-2). When VEGF binds to VEGFR-1, chemotaxis of leukocytes (inflammation) is induced [25]. On the other hand, when VEGF binds to VEGFR-2, the expression of inflammatory factors such as MCP-1 and ICAM-1 is enhanced via nuclear factor-kappa B [13]. Thus, both chemotaxis and leukocyte adhesion to the vascular endothelium are promoted by VEGF, leading to inflammation, which could explain its close relation to the aqueous flare value. However, there was no significant correlation between the aqueous flare value and the vitreous fluid level of sVEGFR-2. We previously reported that the vitreous level of sVEGFR-2 may be regulated independently of VEGF, even though the VEGF-VEGFR-2 signaling pathway has an essential role in regulating vascular permeability [13]. Therefore, sVEGFR-2 may not directly influence the aqueous flare value. VEGF also showed the strongest correlation with the nonperfused area of the retina among the factors investigated. Because VEGF production is upregulated by retinal hypoxia in BRVO patients with macular edema [26], this finding is considered to be reasonable.

The SCORE study [7] recently showed that intravitreal injection of triamcinolone acetonide could improve visual acuity and macular edema in patients with BRVO. Considering this reported efficacy of steroid therapy, a causative role of inflammation is suggested, which is supported by the high aqueous flare values detected in our study. We also found that vitreous fluid levels of VEGF, IL-6, and MCP-1 were significantly correlated with the aqueous flare value and the severity of macular edema in the BRVO patients, indicating that inflammatory cytokines are elevated in patients with higher flare values. Corticosteroids may not only prevent the production of VEGF, but may also suppress various inflammatory molecules (such as IL-6 and MCP-1 [27,28]) that promote leukocyte adhesion along with breakdown of the blood-retinal barrier and thus increase vascular permeability [29]. Therefore, if the aqueous flare value is high, combining anti-VEGF therapy with intravitreal injection of triamcinolone acetonide might be considered.

It was reported that 13 out of 38 patients (34.6%) had persistent macular edema despite receiving a mean of 6.2 injections of bevacizumab [3], while 17 out of 134 patients (12.6%) showed no improvement of visual acuity after monthly intravitreal injection of ranibizumab (0.3 mg) for 6 months [4]. Such patients with persistent macular edema may have low VEGF levels or high levels of other inflammatory factors. It is also possible that patients might have mutation of VEGFR-2 [30,31] and/or one or more of the signal transduction factors [32]. In these patients, it could be worthwhile to examine the severity of inflammation by measuring the aqueous flare value. Our results, taken together with such reports, suggest that inflammatory factors could be a potential target for preventing an increase of vascular permeability in BRVO patients and macular edema. Thus, measuring the aqueous flare value might help to select the treatment strategy for BRVO-associated macular edema. However, a randomized, prospective, clinical trial of anti-VEGF therapy and/or triamcinolone acetonide would be required to demonstrate efficacy for macular edema associated with BRVO.

This study also had some other limitations. For example, the vitreal levels of cytokines may be elevated due to disruption of the blood-retinal barrier. However, we were not able to measure total vitreous protein levels because the vitreous samples were too small. We previously found that the vitreous level of PEDF was decreased in BRVO patients [33], which suggests that levels of cytokines in the vitreous fluid are not determined by disruption of the blood-retinal barrier. In addition, it has been reported that VEGF-A was the only cytokine showing a significant correlation with SD-OCT parameters (thickness of the neurosensory retina and disruption of the ellipsoid line) [34]. However, we could not assess the influence of the outer photoreceptor layer on the visual prognosis because detecting the junction between inner and outer layers was difficult with our OCT equipment. Accordingly, the prognosis of patients with BRVO and macular edema needs to be investigated in more detail in the future.

## Conclusions

We found that the aqueous flare value was significantly higher in BRVO group than in the MH group, that there was a significant correlation between the aqueous flare value and the foveal thickness in the BRVO group, and that vitreous fluid levels of VEGF, IL-6, MCP-1, and sICAM-1 were significantly correlated with both the aqueous flare value and the foveal thickness in the BRVO group. These findings suggest that inflammatory factors (VEGF, IL-6, MCP-1, and sICAM-1) may induce an increase of vascular permeability and disrupt the blood-aqueous barrier in BRVO patients with macular edema.

## Competing interests

No conflicting relationship exists for any author.

## Authors’ contributions

HN was involved in the design and conduct of the study. Collection and management of the data were done by HN, and KS, while analysis and interpretation of the data were performed by HN, TM, and KS. Preparation of the first draft of the manuscript was done by HN, and review and approval of the manuscript was performed by TM, and KS. All authors read and approved the final manuscript.

## Pre-publication history

The pre-publication history for this paper can be accessed here:

http://www.biomedcentral.com/1471-2415/14/67/prepub
